# Proton Pump Inhibitor Use in the U.S. Ambulatory Setting, 2002–2009

**DOI:** 10.1371/journal.pone.0056060

**Published:** 2013-02-13

**Authors:** Stephen R. Rotman, Tara F. Bishop

**Affiliations:** 1 Department of Medicine, New York Presbyterian Hospital, New York, New York, United States of America; 2 Division of Outcomes and Effectiveness, Department of Public Health, Weill Cornell Medical College, New York, New York, United States of America; 3 Department of Medicine, Weill Cornell Medical College, New York, New York, United States of America; University of Colorado, United States of America

## Abstract

**Background and Aims:**

Anecdotal reports and studies of select populations suggest that the use of proton pump inhibitors (PPIs) has increased since their introduction. We sought to determine recent trends in PPI use in the U.S. outpatient setting and characteristics of patients and physicians that may predict their use.

**Methods:**

We used data from the National Ambulatory Medical Care Survey (NAMCS) and the National Hospital Ambulatory Medical Care Survey (NHAMCS) to estimate the prevalence of visits in which patients used PPIs from 2002 to 2009. We tested for associations between PPI use and patient, physician, and practice characteristics using data from 2009. We also estimated the prevalence of visits in which PPIs were used by patients without gastrointestinal complaints, diagnoses, or other indications for their use and tested for associations between patient and physician characteristics and PPI use in patients with no documented indication.

**Results:**

PPIs were used in 4.0% of visits in 2002 and 9.2% in 2009 (p<0.001 for trend across years). The use of omeprazole (0.9% in 2002 to 3.9% in 2009, p<0.001), esomeprazole (0.9% in 2002 to 2.3% in 2009, p<0.001), and pantoprazole (0.6% in 2002 to 1.6% in 2009, p<0.001) increased significantly over the study period. Among visits by patients using PPIs, 62.9% documented no gastrointestinal complaints, gastrointestinal diagnoses, or other indicated reason for their use.

**Conclusions:**

We found that PPI use increased significantly from 2002 to 2009 as did documented indications for their use. Newly-prescribed PPI use did not change from 2006 to 2009. More research is needed to determine whether PPIs are overused in the U.S. outpatient setting.

## Introduction

Overuse of healthcare services is often cited as a driver of rising healthcare costs [1]–[4] and is an indicator of poor quality care [5]–[7]. Anecdotal reports and studies of select populations suggest that the use of proton pump inhibitors (PPIs) has increased since their introduction in the late 1980s [8]–[15].

PPIs are used to treat gastrointestinal conditions such as gastro-esophageal reflux disease (GERD) and peptic ulcer disease (PUD) or in patients who may be at high risk for these diseases (e.g. patients on non-steroidal anti-inflammatories [NSAIDs] and anti-platelet therapy). Although PPIs are generally believed to be safe medications, recent studies indicate that there may be harms associated with their use such as pneumonia and fracture [16]–[25]. Overuse of PPIs may put patients at unnecessary risk for these harms and may also contribute to rising health care costs.

One study has documented increased PPI use in the U.S. outpatient setting but to our knowledge, no studies have examined very recent national trends in PPI use in the U.S. outpatient setting, the characteristics of patients on PPIs, the characteristics of physicians who prescribe PPIs, and trends in indications for their use [13]. Knowledge of these trends and characteristics may inform patients, physicians, payers, and policymakers who want to receive or deliver high quality, high value care.

We used data from two national surveys of visits to ambulatory physicians to describe recent trends in the use of PPIs in the ambulatory setting. We explored potential reasons for these trends by looking at changes in the prevalence of newly prescribed PPIs, changes in histamine blocker (H_2_-blocker) use, and changes in the prevalence of indications for their use.

## Methods

### Source of Data

We used data from the National Ambulatory Medical Care Survey (NAMCS) and the National Hospital Ambulatory Medical Care Survey (NHAMCS) from 2002 through 2009. The NAMCS and NHAMCS are annual surveys conducted by the Center for Disease Control’s (CDC’s) National Center for Health Statistics (NCHS) on a nationally representative sample of visits to physicians in office-based practices and hospital outpatient departments [26]–[27].

The NAMCS and NHAMCS use a three-stage sampling design. The first stage is based on geographic location, the second stage identifies offices in each geographic location, and the third stage samples visits within each office. The visits sampled take place during a one week period that is randomly assigned for each practice. Between 20% and 100% of the visits that week are sampled depending on the size of the practice. The NCHS weighs each visit so that the data can be used for national estimates. Each visit weight accounts for selection probability, adjusts for non-response, and accounts for other factors so that the national estimates properly reflect the scope of ambulatory visits in the U.S. Physicians in the fields of anesthesiology, radiology, and pathology are excluded from the survey. Physicians who participate in the survey cannot participate again for at least three years. There has been no change in the sampling design for our study period.

The surveys collect physician and office demographics, patient demographics, and visit-specific clinical information. For each visit, the surveys record up to three diagnoses based on the *International Classification of Diseases, Ninth Revision, Clinical Modification (ICD-9 CM)* and up to three reasons for visits which are based on the patient’s complaints or symptoms. The surveys also record up to eight medications that the patient is currently taking or that are prescribed at the visit. The survey specifically asks for both prescribed and over-the-counter medications. The information from each visit is recorded on a standardized survey form by the physician, office staff, or a U.S. Census Bureau representative. Each visit is weighted so that national estimates can be calculated. The study was approved by the institutional review board of Weill Cornell Medical College.

### Study Design and Sample

We performed a trend analysis using NAMCS and NHAMCS data from 2002 to 2009 and a cross-sectional analysis using data from 2009. We included all visits by patients 18 years and older who saw a physician.

### Variables

Our main outcome variable was PPI use calculated both as the number and percent of visits in which a PPI was prescribed, ordered, supplied, administered, or continued. PPIs included omeprazole, lansoprazole, rabeprazole, pantoprazole, and esomeprazole (Table 1) [28]. We excluded dexlansoprazole because it was introduced in 2009 and was used in very few visits in that year. To understand whether changes in use could have been due to more new PPI prescriptions, decreased H_2_-blocker use, or more documented indications for PPIs, we also looked at new PPI prescription and overall H_2_-blocker use and documented indications (see below). H_2_-blockers included ranitidine, cimetidine, and famotidine (Table 1) [28].

**Table 1:** Proton pump inhibitors and H_2_-blockers used in the U.S.

Generic Name	Trade Name	Year introduced	Year patent expired	Year available over the counter	Manufacturer
**Proton Pump Inhibitors**					
Omeprazole	Prilosec	1989	2002	2003	Astra Zeneca
Lansoprazole	Prevacid	1995	2009	2009	Takeda Pharm
Rabeprazole	Aciphex	1999	2006	NA	Eisai Inc. and Janssen
Pantoprazole	Protonix	2000	2006	NA	Wyeth Pharm
Esomeprazole	Nexium	2001	2008	NA	Astra Zeneca
Dexlansoprazole	Dexilant	2009	NA	NA	Janssen-Cilag
**H^2^-blockers**					
Cimetidine	Tagamet	1977	1994	1996	GlaxoSmithKline
Ranitidine	Zantac	1983	1995	1995	GlaxoSmithKline
Famotidine	Pepcid	1987	2000	1995	Merck, Johnson & Johnson
Nizatidine	Axid	1988	2002	1996	Braintree Laboratories

Our main predictor variables were year (2002 to 2009), patient age, patient gender, patient race/ethnicity (white, black, Hispanic, other), number of chronic medical conditions (0, 1–3, >3), primary payer (private insurance, Medicare, Medicaid, other), physician specialty category (primary care, medical specialist, surgical specialist), and practice type (private practice, community health center, health maintenance organization, hospital outpatient department, other).

We defined an indication for PPI use as a visit in which a gastrointestinal diagnosis (GERD, gastrointestinal ulcer, gastrointestinal bleed, esophagitis, Barrett’s esophagus, esophageal varices, dyspepsia, gastritis, Helicobacter Pylori infection, malignant neoplasm of the esophagus or stomach, hepatitis, or liver disease) was documented, the patient reported a gastrointestinal complaint or a potential symptom that may be caused by a gastrointestinal diagnosis (gastrointestinal bleeding, heartburn, abdominal pain, chest pain, or cough), or documentation of a medication where prophylaxis might be necessary (NSAIDs, anti-platelet therapies, steroids) [29].

### Analysis

We performed a visit-level analysis using visit sampling weights to account for clustering at the physician and practice level and to generate national estimates of counts and percentages. We used the Pearson chi-squared test to compare patient and physician characteristics between 2002–2003 and 2008–2009. We used linear regression to test for linear trends in PPI use (overall and for specific PPIs) between 2002 and 2009 while controlling for patient and physician characteristics (age, gender, payer, race/ethnicity, type of practice).

We used the Pearson chi-squared test and multivariable logistic regression to test for associations between PPI use and patient, physician, and practice characteristics using data from 2008 and 2009. We also estimated the prevalence of visits in which PPIs were used by patients without gastrointestinal diagnoses, complaints, or concomitant high-risk medication use. We used multivariable logistic regression to test for associations between the patient and physician characteristics described above and PPI use in patients with no documented indication. All tests were two-sided with a p-value of 0.05 considered significant.

## Results

There were approximately 772 million ambulatory visits by adults patients in 2002. The number of ambulatory visits increased to 919 million visits in 2009. Patients seen in 2008–2009 were older than patients seen in 2002–2003 (47.6% ≥65 years in 2008–2009 vs. 40.5≥65 years in 2002–2003), their primary payer was more likely to be Medicare (46.5% in 2008–2009 vs. 36.0% in 2002–2003), and were more likely to be see in a community health center (2.7% in 2008–2009 vs. 0.2% in 2002–2003) (Table 2).

**Table 2:** Characteristics of visits, 2002–2003 and 2008–2009^a^.

	Visits in 2002–2003, %	Visits in 2008–2009,%	p-value
Age			**0.001**
18–29	4.2	3.0	
30–49	25.8	19.2	
50–64	29.5	30.2	
65–79	27.1	33.4	
≥80	13.4	14.2	
Female Gender	61.7	62.5	0.68
Race/Ethnicity			
White	76.5	75.8	0.91
Black	10.0	9.9	
Hispanic	9.2	10.4	
Other	4.3	3.8	
No. Chronic Conditions^b^			
0	–	17.5	–
1–3	–	66.0	
>3	–	16.6	
Primary Payer			
Private	49.2	41.0	**<0.001**
Medicare	36.0	46.5	
Medicaid	8.5	6.0	
Other	6.3	6.5	
MD Specialty			
Primary Care	–	55.0	–
Medical Specialist	–	34.2	
Surgeon	–	10.8	
Practice Type			
Private Practice	84.0	84.2	**<0.001**
CHC	0.2	2.7	
HMO	1.1	1.1	
Hospital OPD	7.5	8.8	
Other	7.3	3.2	

a Weighted percentages based on the sample that was surveyed.

b Variable not available in the 2002 and 2003 databases.

**Figure 1:**
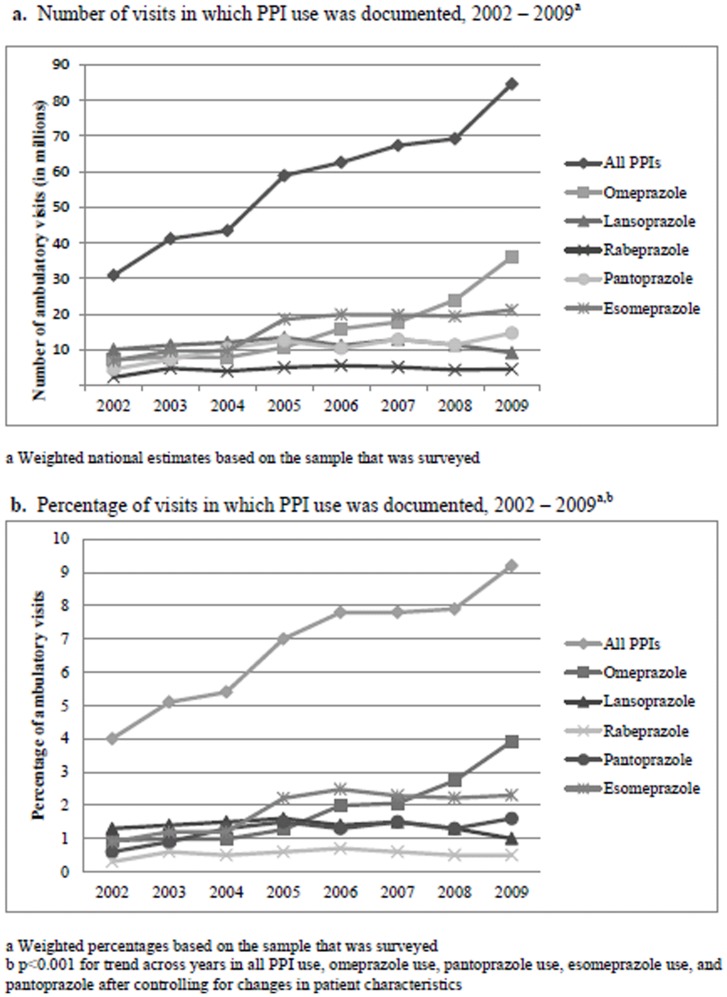
Changes in PPI use, 2002–2009.

The number of visits with documented PPI use increased from 30 million visits in 2002 to 84 million visits in 2009 (Figure 1A). Overall PPI use rose significantly from 2002 to 2009 (4.0% of visits in 2002 to 9.2% of visits in 2009, p<0.001, Figure 1B) even after controlling for patient, physician, and practice demographic changes. We found significant increases in the use of omeprazole (0.9% of visits in 2002 to 3.9% of visits in 2009, p<0.001), esomeprazole (0.9% of visits in 2002 to 2.3% of visits in 2009, p<0.001), and pantoprazole (0.6% of visits in 2002 to 1.6% of visits in 2009, p<0.001). These findings were, again, significant even after controlling for patients, physician, and practice demographic changes.

In 2008 and 2009, patients on PPIs were older than patients not on PPIs (46.7% of patients on PPIs were >65 vs. 30.3% of patients not on PPIs, p<0.001) and were more likely to have one or more chronic medical conditions (81.9% vs. 64.3%, p<0.001) (Table 3). Patients on PPIs were more likely to be seeing a primary care physician (55.0% vs. 51.3%, p<0.001) or medical specialist (34.2% vs. 27.1%, p<0.001) than a surgeon (10.8% vs. 21.6%, p<0.001).

**Table 3:** Characteristics of visits by patients on PPIs, 2008–2009^a^.

	No PPI, %	On PPI, %	p-value
Age			**<0.001**
18–29	12.3	3.4	
30–49	29.0	20.6	
50–64	28.5	29.3	
65–79	21.4	32.6	
≥80	8.9	14.1	
Female Gender	61.8	60.8	0.50
Race/Ethnicity			
White	74.3	78.5	0.06
Black	11.5	9.3	
Hispanic	10.2	9.0	
Other	4.0	3.2	
No. Chronic Conditions			**<0.001**
0	35.7	18.1	
1–3	55.1	63.9	
>3	9.2	18.0	
Primary Payer			**<0.001**
Private	53.4	42.1	
Medicare	29.2	46.2	
Medicaid	8.5	6.3	
Other	9.0	5.4	
MD Specialty			**<0.001**
Primary Care	51.3	55.0	
Medical Specialist	27.1	34.2	
Surgeon	21.6	10.8	
Practice Type			
Private Practice	83.6	84.7	0.20
CHC	2.5	3.5	
HMO	1.4	0.6	
Hospital OPD	8.0	7.6	
Other	4.6	3.6	

a Weighted percentages based on the sample that was surveyed.

**Figure 2:**
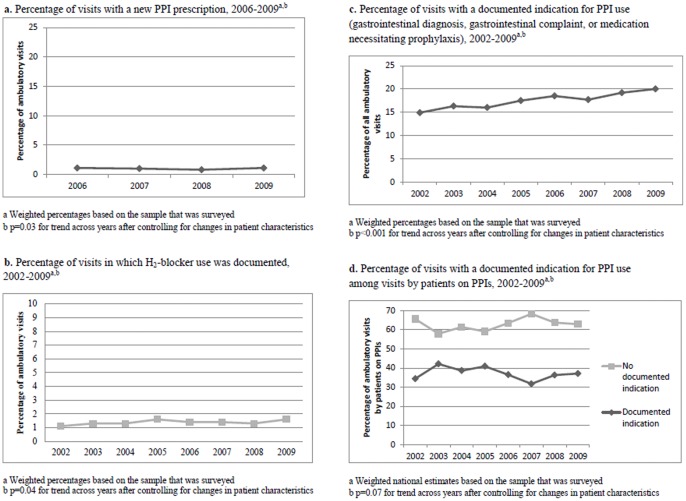
Changes in possible reasons for increased PPI use.

From 2006 to 2009, the percentage of newly prescribed PPIs was very small and did not increase (1.1% of visits in 2006 vs. 1.1% of visits in 2009, p = 0.03, Figure 2A). From 2002 to 2009, H_2_-blocker use did not decrease and, in fact, increased a small but significant amount (1.1% of visits in 2002 to 1.6% of visits in 2009, p = 0.04, Figure 2B). Possible indications for their use among all visits increased from 2002 to 2009 (14.9% of visits in 2001 to 20.0% of visits in 2009, p<0.001) but the percentage of visits with a documented indication for their use among patients on PPIs did not change (34.4% of visits in 2002 to 37.1% of visits in 2009, p = 0.07).

In all years, the majority of visits with documented PPI use had no documented indication (Figure 2B). Among visits by patients using PPIs in 2009, 62.9% of patients had no documented gastrointestinal complaints, gastrointestinal diagnoses, or concomitant high-risk medication. In multivariable analyses, we found no correlation between any physician or practice characteristics and PPI use without a documented indication (Table 4).

**Table 4:** Association between patient and physician characteristics and use of PPIs in patients with no documented indication, 2009a,b.

	PPI use with no documented indication			
	No, %	Yes, %	aOR^b^	Adjusted p-value^b^
Age				
18–29	3.4	3.5	Ref	
30–49	20.2	21.3	1.02	0.94
50–64	29.9	28.2	0.88	0.76
65–79	32.5	32.9	1.06	0.88
≥80	14.1	14.1	1.07	0.87
Gender				
Female	62.1	58.6	Ref	
Male	37.9	41.4	1.14	0.30
Race/Ethnicity				
White	78.6	78.3	Ref	
Black	9.1	9.7	1.16	0.57
Hispanic	9.3	8.4	0.91	0.60
Other	3.0	3.6	1.49	0.38
No. Chronic Conditions				
0	17.6	18.9	Ref	
1–3	63.4	64.8	0.99	0.98
>3	19.0	16.4	0.85	0.54
Primary Payer				
Private	41.5	43.1	Ref	
Medicare	46.4	45.7	0.90	0.55
Medicaid	6.5	6.0	1.04	0.89
Other	5.5	5.2	1.11	0.72
MD Specialty				
Primary Care	56.2	53.0	Ref	
Medical Specialist	11.8	9.1	7.66	0.12
Surgeon	32.1	37.9	1.24	0.17
Practice Type				
Private Practice	84.0	85.9	Ref	
CHC	4.3	2.1	1.24	0.17
HMO	0.6	0.5	0.54	0.12
Hospital OPD	7.7	7.6	Omitted	–
Other	3.4	3.9	1.15	0.67

a Weighted percentages based on the sample that was surveyed.

b Adjusted Odds Ratio controlling for patient age category, patient gender, patient race/ethnicity, primary payer, physician specialty, and practice type.

## Discussion

In this study of PPI use in the ambulatory setting, we found almost a three-fold increase in their use in recent years. In 2009, PPI use was documented in almost a tenth of ambulatory visits (over 80 million visits) compared with close to 4 percent of visits in 2002.

We explored three potential reasons for increased use of PPIs: continuation of previously prescribed PPIs, a shift to use PPIs rather than other acid reducers such as H_2_-blockers, and more reasons for their use because of gastrointestinal diagnoses, patient symptoms, and medications.

Our finding of little change in new prescriptions for PPIs suggests that patients stay on PPIs chronically, that they may be started in settings other than the outpatient setting (e.g. hospital, nursing home), or that self-prescribe over-the-counter PPIs. The second explanation is not supported by our findings: H_2_-blocker use did not decrease over the study period and, in fact, increased over our study period. The third explanation, increased documented indications, may also contribute to increased PPI use over the study period.

Nevertheless, in all study years, we found that the majority of visits with documented PPI use had no documented indication for their use. These findings raise the question of whether PPI use since 2002 reflects overuse rather than appropriate use. Potential reasons for overuse include PPI continuation after a short term indication (e.g. hospitalization), a belief that PPIs offer benefit with little harm, and aggressive marketing to patients and physicians.

Interestingly, the two individual PPIs with the most significant increase in their use were omeprazole and esomeprazole. Both of these medications are made by the same manufacturer (Astra Zeneca) and their increased use may reflect effective marketing - both medications have been marketed as “purple pills” in multiple media setting [30]. However, this may be mere coincidence particularly because esomeprazole is not the most frequently prescribed PPI. Increased omeprazole use may also be the result of increased availability as an over-the-counter medication, its long time on the market, and its availability in generic formulations [28].

Our findings are in concert with reports that PPI use is increasing worldwide. Reports from Taiwan, the United Kingdom, and Australia have all documented increased use [10], [14], [15]. For example, in Australia, researchers found a greater than one thousand-fold increase in PPI use from 1995 to 2006 [9]; in the United Kingdom researchers have documented that a majority of PPIs are prescribed inappropriately [14].

Unfortunately, recent work has elucidated potential harms of PPIs including pneumonia, fracture, enteric infection, and malabsorption [16]–[25]. One study found a 1.6 fold increased risk of community acquired pneumonia in patients on PPIs [22]. Another found a 1.3 fold increased odds of hospital-acquired pneumonia in patients on PPIs [19]. Analyses of data from the United Kingdom showed a 1.5 fold increased risk of hip fracture with long-term PPI use [24].

Further, literature also suggests that the benefits of PPIs may be overstated particularly for prophylaxis in hospitalized patients [25]. In fact, a recent literature review found no significant difference in stress ulcer prevalence in hospitalized patients who received H_2_-blockers and PPIs [21]. If, in fact, such a high percentage of patients are on PPIs for no reason, we may be putting patients at undue risk.

Our study is limited primarily by the data available through the NAMCS and NHAMCS. First, our evaluation is at the visit level, not at the patient level so the percentages we report of percent of visits, not percent of patients. It is possible that there is not a direct correlation between the number of patients on PPIs and their use documented at the visit level or it is possible that patients on PPIs have more visits than patients not on PPIs. We did, however, look at trends across years and documented medication use, diagnoses, and symptoms at the visit level for multiple years. Second, our data are limited to what is documented from the patient record. Although the surveys do ask for over-the-counter medications, it is possible that PPIs that are available over-the-counter may not be documented in the patient record. Conversely, we may be overestimating potentially inappropriately used PPIs because not all symptoms, diagnoses, and medications are documented in NAMCS and NHAMCS. We also do not know whether PPIs were prescribed on an as needed basis (prn) or the duration of therapy. Lastly, it is possible that patients remain on PPIs long-term because of rebound symptoms when they are removed from PPIs [31].

In summary, we found a large and significant increase in PPI use in the U.S. outpatient setting since 2002 but no increase in PPI use without a documented indication or in new PPI prescriptions. Nevertheless, the majority of patients on PPIs in all years had no documented indication. Our findings confirm what has been documented in smaller settings, older studies and international settings. Our findings suggest that inappropriate PPI use is not necessarily increasing but is still an important public health problem.

While growing evidence points out important adverse associations with PPIs, they do remain effective drugs for their specified indications. More research is needed to fully understand the scope of overuse of PPIs in the ambulatory setting. These methods include more granular reviews of their use in the ambulatory setting or studies to understand why physicians prescribe and patients use PPIs when the indications are not clear. Further research should also address methods to change physician and patient decisions regarding their use. Interventions such as education, treatment guidelines, and decision support systems may address this problem. Ultimately, however, physicians, payers, policymakers, and even patients should be tasked with evaluating the need for PPI therapy, especially for long-term use.
